# *De novo *assembly and transcriptome analysis of five major tissues of *Jatropha curcas *L. using GS FLX titanium platform of 454 pyrosequencing

**DOI:** 10.1186/1471-2164-12-191

**Published:** 2011-04-15

**Authors:** Purushothaman Natarajan, Madasamy Parani

**Affiliations:** 1 Genomics Laboratory, Department of Genetic Engineering, SRM University, Chennai, Tamil Nadu, 603 203, India

## Abstract

**Background:**

*Jatropha curcas *L. is an important non-edible oilseed crop with promising future in biodiesel production. However, factors like oil yield, oil composition, toxic compounds in oil cake, pests and diseases limit its commercial potential. Well established genetic engineering methods using cloned genes could be used to address these limitations. Earlier, 10,983 unigenes from Sanger sequencing of ESTs, and 3,484 unique assembled transcripts from 454 pyrosequencing of uncloned cDNAs were reported. In order to expedite the process of gene discovery, we have undertaken 454 pyrosequencing of normalized cDNAs prepared from roots, mature leaves, flowers, developing seeds, and embryos of *J. curcas*.

**Results:**

From 383,918 raw reads, we obtained 381,957 quality-filtered and trimmed reads that are suitable for the assembly of transcript sequences. *De novo *contig assembly of these reads generated 17,457 assembled transcripts (contigs) and 54,002 singletons. Average length of the assembled transcripts was 916 bp. About 30% of the transcripts were longer than 1000 bases, and the size of the longest transcript was 7,173 bases. BLASTX analysis revealed that 2,589 of these transcripts are full-length. The assembled transcripts were validated by RT-PCR analysis of 28 transcripts. The results showed that the transcripts were correctly assembled and represent actively expressed genes. KEGG pathway mapping showed that 2,320 transcripts are related to major biochemical pathways including the oil biosynthesis pathway. Overall, the current study reports 14,327 new assembled transcripts which included 2589 full-length transcripts and 27 transcripts that are directly involved in oil biosynthesis.

**Conclusion:**

The large number of transcripts reported in the current study together with existing ESTs and transcript sequences will serve as an invaluable genetic resource for crop improvement in jatropha. Sequence information of those genes that are involved in oil biosynthesis could be used for metabolic engineering of jatropha to increase oil content, and to modify oil composition.

## Background

The genus Jatropha of Euphorbiaceae family contains about 175 known species [1] of which *Jatropha curcas *L. is the most promising and suitable species for biodiesel production worldwide. Biodiesel is produced by transesterification of the oil to fatty acid methyl esters. Being non-edible crop, use of jatropha oil for biodiesel production does not threaten food security. Its cultivation in degraded soil can control erosion, and help in land reclamation. It is used for manufacturing soap, purgative agents, candles, coloring dyes and astringents [2]. Medicinal properties of jatropha include anti-tumor, anti-microbial, anti-parasitic, and anti-diarrheal activities [3-6].

The oil content of Jatropha which is around 30% could be increased to about 50% in order to make it commercially viable. In terms of oil composition, decreasing the content of unsaturated fatty acids (to increase oxidative stability), free fatty acids (to prevent soap formation and to increase the yield of biodiesel), and 18-carbon fatty acids (to lower the viscosity for better atomization of the biodiesel) would help to improve the quality of jatropha biodiesel. Jatropha oil cake - the by product of oil extraction, is not used as animal feed due to the presence of toxic curcin and phorbol esters. Since it is not economical to remove these compounds, it would be desirable to block their accumulation itself by seed-specific silencing of the relevant genes. This will add economic value to the crop. Large scale cultivation of jatropha also requires the development of varieties that are tolerant to drought, and resistant to pests and diseases.

Genetic engineering methods could play a major role in jatropha crop improvement, because the scope for classical breeding is limited due to longer breeding cycle. Genetic manipulations in jatropha require genomic information and cloning of all the important genes. The genome size of *J. curcas *is estimated to be 410Mb [7]. The jatropha genome has been fully sequenced by Synthetic Genomics Inc, California, USA but it is not available for public use [8]. Partial genomic sequence of 285.8Mb is available in public databases but it is not assembled and annotated [9]. Natarajan *et al.*, 2010 [10] have reported 12,084 ESTs using a normalized cDNA library from developing seeds, and Costa *et al.*, 2010 [11] have reported 13,249 ESTs using non-normalized cDNA libraries from developing and germinating endosperm. Contig assembly of these ESTs showed the presence of 10,983 unigenes (6,361 from Natarajan *et al.*, 2010 [10] and 4,622 from Costa *et al.*, 2010 [11]). Recently, Sato *et al.*, 2011 [9] have reported 991,050 pyrosequencing reads from leaf and callus transcriptome which were assembled in to 4,751 contigs. Hybrid assembly of the unigenes and the assembled transcripts revealed the presence of 14,467 unique sequences (10,983 unigenes and 3,484 unique assembled transcripts). This is far below the number of sequences required to represent the whole transcriptome of jatropha. Annotation of the currently available unique sequences showed that several important genes are yet to be cloned.

High throughput 454 pyrosequencing of uncloned cDNAs is a powerful method for whole genome transcriptome analysis and gene discovery. 454 pyrosequencing using GS FLX platform was done in many plants including arabidopsis [12], artemisia [13], cucumber [14], medicago [15], maize [16] and barley [17], and an average read length of 100-200 bases was obtained. Shorter read length was a major problem in assembly, especially in case of *de novo *assembly of the data from novel organisms, which do not have previously assembled and annotated reference sequences [18,19]. This limitation has been quickly overcome by using the newly launched titanium platform of GS FLX which increased the average read length to as high as 422 bases [20]. Concurrent improvements in *de novo *assembly software made it possible to assemble large number of full-length transcripts from novel organisms using 454 pyrosequencing data. Here, we report 454 pyrosequencing of normalized cDNAs from roots, mature leaves, flowers, developing seeds, and mature embryo of *J. curcas *using GS FLX titanium platform, and report *de novo *assembly of 17,457 transcripts.

## Results and Discussion

### cDNA synthesis and normalization

Total RNA was isolated from roots, mature leaves, flowers, developing seeds, and embryos of *J. curcas*. Quality of the RNA as determined by agarose gel electrophoresis (additional file 1) and OD_260_/OD_280 _ratio (1.9 ± 0.05) was found to be suitable for cDNA synthesis. The total RNA from the five tissues were pooled and normalized cDNA was synthesised. Normalization of the cDNA greatly reduces the frequency of abundant transcripts, and increases the rate recovery of unique transcripts [10]. The efficiency of normalization was monitored by doing a parallel normalization reaction using chloramphenicol resistance gene as reporter gene. Redundant rate of the reporter gene was reduced from 1% to 0.025% after normalization which indicated 40 fold reduction in abundance. The normalized cDNA was subjected to quality control experiments before using it for 454 pyrosequencing. A test cDNA library was constructed using an aliquote of the normalized cDNA, and clones were randomly selected for further analysis. The average size of the normalized cDNAs as determined by PCR was about 2.3 kb (additional file 1) which is higher than average transcript size. Sequencing and BLASTX analysis revealed that 90% of the clones were full-length at the 5' end. These results showed that the normalized cDNA is highly suitable for 454 pyrosequencing.

### 454 pyrosequencing using GS FLX titanium platform

**Figure 1:**
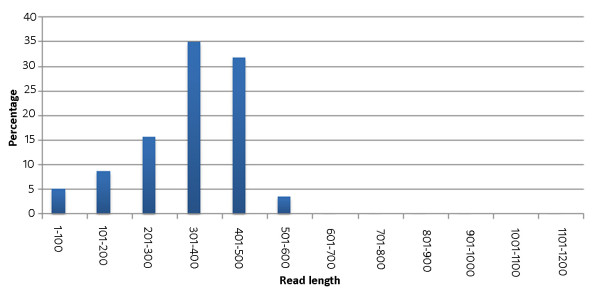
**Size distribution of the 454 pyrosequencing reads after quality filtering and removal of primer and adapter sequences**.

Half-plate 454 pyrosequencing reaction of the normalized cDNA was done using GS FLX titanium platform. It produced 197.7 Mb data from 383,918 reads with an average read length of 515 bases (53 to 1201 bases). First, the sequences were filtered using a minimum quality cut off value of 40. Then, the additional sequences that were added during cDNA synthesis were trimmed off. Poly (A/T) sequences were retained as they were reported to be useful in transcript assembly [21]. Sequences with less than 50 bases were removed before assembly. Finally, 381,957 ready-to-assemble reads were obtained. Size distribution of these reads is shown in figure 1. Length of these reads ranged between 50 and 1169 bases with an average of 343 bases.

### *De novo *assembly

Reference assembly with the partial genomic sequence of jatropha [9] showed mapping of 95.87% of the reads, and the consensus accuracy was 99.15%. The details of reference assembly are given in additional file 2. However, it could not be used for transcript assembly because the genome is not assembled and annotated. Therefore, *de novo *assembly was done using GS *de novo *assembler. This assembler can assemble the data under genomic or cDNA option. The genomic option assembles overlapping reads and reports consensus sequences as contigs. The cDNA option continues with linking the contigs together to form isotigs (splice variants) and isogroups (all the splice variants of individual transcripts). Since the objective of this study was only to assemble the transcripts, the genomic option was used. GS *de novo *assembler version 2.5p1 was reported to be better than the version 2.3 for *de novo *assembly using cDNA option [21]. Therefore, we have evaluated the currently available three versions of the assembler (v.2.3, v.2.5p1 and v.2.5.2) using genomic option. Contig assembly was done under the stringent assembly parameters of 40 bases overlap and 95% overlap identity. The results did not show significant differences among the versions in terms of the number of reads assembled into contigs, number of contigs assembled, average contig length, and the length of the longest contig (Table 1). Therefore, we have used the output from the latest version of the assembler (v.2.5.2) for further analysis.

**Table 1:** Comparison of the performance of the three versions of GS *de novo *assemblers for genomic assembly

Description	Version 2.3	Version 2.5p1	Version 2.5.2
No. of reads used for assembly	304,619	303,601	303,601
No. of reads assembled in to contigs	248,857	247,757	247,757
No. of contigs assembled	17, 613	17,457	17,457
No. of singletons	54,669	54,002	54,002
Average contig length (bases)	909	916	916
Largest contig length (bases)	7,170	7,173	7,173

**Figure 2:**
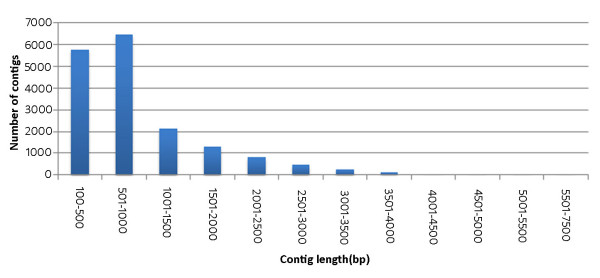
**Size distribution of the contigs generated by *de novo *assembly of the quality filtered and trimmed 454 pyrosequencing reads using GS *de novo *assembler version 2.5.2**.

The number of ESTs that are available from jatropha based on Sanger sequencing is 25,333 [10,11]. From the first 454 pyrosequencing study in jatropha, 991,050 reads with an average read length of 407 bases were reported by Sato *et al.*, 2011 [9]. Analysis of these reads showed the presence of 822,387 repeat reads (83%), thereby, reducing the effective number of reads to 168,663. We have obtained 381,957 reads with an average read length of 343 bases, and there were only 36 repeat reads (0.01%). This may be because we have used cDNAs that were normalized against abundant transcripts. In addition, we have used a more diverse pool of cDNAs from five different tissues. These two factors have also played a role in the number of contigs assembled and the contig length. Contig assembly showed 4,751 contigs with an average contig length of 749 bases, and 17,457 contigs with an average contig length of 916 bases from the effective reads of Sato *et al.*, 2011 [9] and the current study, respectively. The sequences of the assembled contigs are given in additional file 3 and 4. The size distribution of the contigs obtained from the current study is shown in figure 2. Hybrid assembly of the existing ESTs [10,11] and assembled contigs [9] revealed the presence of 10,983 unique ESTs from Sanger sequencing, and 3,484 unique transcripts from 454 pyrosequencing. Similar analysis of the 17,457 contigs that were assembled in the present study uncovered 14,327 unique transcripts. Together, at least partial sequences of 28,794 expressed genes are currently known from jatropha.

### Annotation

Similarity search for all the contigs was done against non-redundant protein sequences database (nr) using BLASTX, and the 15,925 contigs that showed significant similarity (e-value cut off of <10^-10^) were annotated. It was found that 2,589 annotated contigs were full-length, and their size ranged from 247 to 5,465 bp. These sequence information could be used for direct amplification of the desired genes. The remaining 13,336 contigs were partial at 3' end (3,889 contigs) or 5' end (3,980 contigs) or both ends (5,467 contigs). These sequence information will be useful in cloning full-length genes using methods like rapid amplification of cDNA ends (RACE) PCR. However, these methods are cumbersome, and are not amenable for high throughput applications. Therefore, it would be easier to increase the number of full-length transcripts by increasing the number of reads from 454 pyrosequencing.

### Pathway mapping of contigs by KEGG

Ortholog assignment and mapping of the contigs to the biological pathways were performed using KEGG automatic annotation server (KAAS). All the contigs were compared against the KEGG database using BLASTX with threshold bit-score value of 60 (default). It assigned EC numbers for 2,320 contigs, and they were mapped to respective pathways. The mapped contigs represented metabolic pathways of major biomolecules such as carbohydrates, lipids, nucleotides, amino acids, glycans, cofactors, vitamins, terpenoids, polyketides, etc. The KEGG pathway analysis also showed that 549 and 241 contigs represent the major metabolic pathways and biosynthetic pathways of secondary metabolites, respectively. The mapped contigs also represented the genes involved in genetic information processing (transcription, translation, folding, sorting and degradation, replication and repair, RNA family), environmental information processing (membrane transport, signal transduction, signaling molecules and interaction) and cellular processes (transport and catabolism, cell motility, cell growth and death, cell communication).

### Genes involved in oil biosynthesis

**Figure 3:**
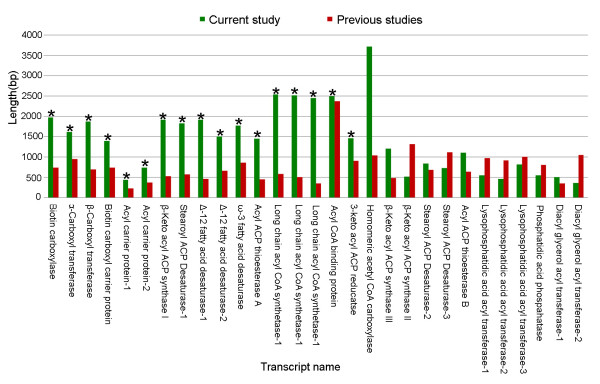
**Comparison of the length of the 28 transcript sequences (involved in oil biosynthesis) those were common between the current study and the previous studies**. (*) Full-length transcripts that were obtained from the current study

Jatropha is popularly grown for biodiesel production. However, making it an ideal biodiesel crop requires genetic manipulations for increased oil yield and modified oil composition using the genes that are involved in oil biosynthesis pathway. Therefore, the contigs that were mapped to oil biosynthesis pathway were analyzed in detail. Biosynthesis of oil (triacyl glycerol) in plants takes place in three major steps, (a) biosynthesis of fatty acids in plastids, (b) activation and transport of fatty acids to endoplasmic reticulum and (c) synthesis of triacyl glycerol or oil. In Arabidopsis, 36 enzymes and 2 proteins are involved in oil biosynthesis [22]. Twenty-eight of these enzymes are encoded by single genes, and the remaining 8 enzymes and the 2 proteins are encoded by small gene families. In Jatropha, 29 non-redundant sequences coding for 20 enzymes and 2 proteins that are involved in oil biosynthesis were already reported [9-11] but all of them were partial sequences. From this study, full-length transcripts were obtained for 17 of these partial sequences and significantly longer transcripts were obtained for another 5 partial sequences (Figure 3**)**. In addition, 11 full-length and 16 partial transcripts coding for 15 enzymes and 1 protein were also identified. In total, 56 sequences related to oil biosynthesis were found from this study, and 27 of them are reported for the first time in jatropha (Table 2).

**Table 2:** Contigs representing the enzymes and proteins involved in oil biosynthesis

S.No	Annotation	No. of genes	Contig Number	Size (bp)	Functions
1	Pyruvate kinase	2	2179, 2717	2013*, 1637	Conversion of phosphoenol pyruvate to pyruvate
2	Pyruvate dehydrogenase E1a subunit	2	2503, 3695	1707*, 1317*	Conversion of pyruvate to acetyl-CoA
3	Pyruvate dehydrogenase E1b subunit	2	1782, 4154	1965*, 1203	Conversion of pyruvate to acetyl-CoA
4	Pyruvate dehydrogenase E2 subunit	3	1049, 1574, 11093	2430*, 2115, 517	Conversion of pyruvate to acetyl-CoA
5	Pyruvate dehydrogenase E3 subunit	2	1908, 3744	1934*, 1299	Conversion of pyruvate to acetyl-CoA
6	Lipoate synthase	1	10908	529	Conversion of pyruvate to acetyl-CoA
7	Lipoyl transferase	1	6252	864	Conversion of pyruvate to acetyl-CoA
8	Homomeric acetyl-CoA carboxylase	1	124	3711	Conversion of acetyl-CoA to malonyl-CoA
9	Heteromeric acetyl-CoA carboxylase biotin carboxylase subunit	1	1836	1972*	Conversion of acetyl-CoA to malonyl-CoA
10	Heteromeric acetyl-CoA carboxylase carboxyl transferase alpha subunit	1	2789	1609*	Conversion of acetyl-CoA to malonyl-CoA
11	Heteromeric acetyl-CoA carboxylase carboxyl transferase beta subunit	1	2079	1868*	Conversion of acetyl-CoA to malonyl-CoA
12	Heteromeric acetyl-CoA carboxylase biotin carboxyl carrier protein	2	3458, 4048	1388*,1232*	Conversion of acetyl-CoA to malonyl-CoA
13	Acyl carrier protein	2	13541,7450	437*,738*	Conversion of malonyl-CoA to malonyl-ACP
14	Beta-ketoacyl-ACP synthase III	1	4145	1204	Conversion of malony-ACP to 3-keto acyl ACP
15	3-Ketoacyl-ACP reductase	1	6732	1455*	Conversion of 3-keto acyl ACP to 3-hydroxy acyl ACP
16	Beta-ketoacyl-ACP synthase I	1	1954	1915*	Serial conversion of butyryl-ACP to palmitoyl-ACP
17	Beta-ketoacyl-ACP synthase II	1	11467	510	Conversion of palmitoyl ACP to Stearoyl-ACP
18	Stearoyl-ACP desaturase	4	6477,7609,2187, 11778	840, 727,1823*,496	Conversion of Stearoyl-ACP to oleoyl-ACP
19	Delta-12 fatty acid desaturase	2	1974, 3130	1914*, 1508*	Conversion of oleoyl-ACP to linoleoyl-ACP
20	Omega-3 fatty acid desaturase	2	1744,1847	2015*,1767*	Conversion of linoleoyl-ACP to α-linolenyl-ACP
21	Fatty acyl-ACP thioesterase B	1	4664	1098	Conversion of saturated fatty acyl-ACP to saturated free fatty acid
22	Palmitoyl-ACP thioesterase	1	7152	769	Hydrolysis of Palmitoyl-ACP to palmitic acid
23	Fatty acyl-ACP thioesterase A	1	5723	1452*	Conversion of unsaturated fatty acyl-ACP to unsaturated free fatty acid
24	Long chain acyl CoA synthetase	4	910,932,1027, 9748	2539*,2516*, 2442*,579	Activation free fatty acid to fatty acyl-CoA
25	Acyl-CoA binding protein	5	3898,955,6476, 13592, 10829	1267,2497*, 840, 436, 524*	Transport of acyl-CoA to the ER
26	Glycerol-3-phosphate acyl transferase	3	4004, 7597, 8581	1244,729,643	Acylation of glycerol-3-phosphate to lysophosphatidic acid
27	Lysophosphatidic acid acyl transferase	5	10599,6878, 11276,12982,6748	543, 799, 511, 462, 811	Acylation of lysophosphatidic acid to phosphatidic acid
28	Phosphatidic acid phosphatase	1	10375	549	Conversion of phosphatidic acid to diacyl glycerol
29	Diacyl glycerol acyl transferase	2	11532,14284	509,361	Acylation of diacyl glycerol to triacyl glycerol
	**Total number of genes**	**56**			

* Contigs representing full length genes

### Biosynthesis of fatty acids

Oxidative decarboxylation of pyruvate to acety-CoA by pyruvate dehydrogenase (PDH) is the first committing step in fatty acid biosynthesis. PDH is a four subunit enzyme complex having pyruvate decarboxylase (E1-*α *and E1-*β *subunits), dihydrolipoyl acetyltransferase (E2 subunit) and dihydrolipoamide dehydrogenase (E3 subunit) enzymes [23]. Contigs representing all the four subunits were identified from the current study. Fatty acid biosynthesis is initiated by the condensation of acetyl-CoA to malonyl-CoA which is catalyzed by heteromeric acetyl CoA carboxylase (ACCase). It consists of three nuclear encoded subunits (biotin carboxyl carrier protein, biotin carboxylase and carboxyl transferase α-subunit) and a plastid encoded subunit (carboxyl transferase β-subunit) [24]. Accumulation of the mRNAs for these four subunits is co-ordinately regulated so that a constant molar stoichiometric ratio is maintained [25]. However, the plastid encoded subunit seems to be crucial for the accumulation of heteromeric ACCase, and for increasing the oil content [26,27]. Plants also have a homomeric ACCase localized in cytosol that does not play any major role in fatty acid biosynthesis that takes place almost exclusively in plastids. However, by targeting this enzyme to plastids, oil content was increased five-fold in potato tubers [28] and 5% in rapeseed [29]. We have found full-length contigs for all the four subunits of heteromeric ACCase and a 5' partial contig (3,711 bases) for homomeric ACCase.

Condensation of malonyl-CoA and acyl carrier protein (ACP) to form 3-carbon malonyl-ACP is catalysed by malony CoA acyl transferase. Polymerization of the fatty acid carbon chains starts with the formation of a 4-carbon butyryl-ACP by the addition of a carbon from acetyl-CoA to the 3-carbon malonyl-ACP by the action of keto acyl ACP synthase III (KASIII). Further chain elongation up to 16-cabon palmitoyl-ACP is carried out by keto acyl ACP synthase I (KASI) in six consecutive steps by adding two carbons in each step. Finally, keto acyl ACP synthase II (KASII) converts the 16-cabon palmitoyl-ACP to 18-carbon stearoyl-ACP. While KASI is essential for seed oil content [30], KASII is useful for manipulating the chain length of the fatty acids. Longer the chain length of fatty acids more is the viscosity of the oil as well as the biodiesel derived from it [31]. Viscosity affects atomization of the biodiesel and lowers the performance of the diesel engines due to engine deposits [32]. Viscosity of jatropha oil can be lowered by reducing its 18-carbon fatty acid content which is reported to be as high as 84.7% [33]. Previous reports suggest that this can be achieved by silencing the activity of KASII that converts the palmitoyl-ACP to stearoyl-ACP [34,35]. Alternatively, the activity of palmitoyl-ACP thioesterase (encoded by FATA1) could be enhanced to accelerate the cleavage of palmitic acid from the palmitoyl-ACP before it is converted to stearoyl-ACP [36]. The contigs for all these genes were found in the current study.

Saturated fatty acids are desaturated to produce unsaturated fatty acids, and plant oils contain both of them in varying proportions. In plants, 18:0 stearoyl-ACP is sequentially desaturated to 18:1 oleoyl-ACP, 18:2 linoleoyl-ACP and 18:3 linolenyl-ACP by Stearoyl ACP desaturase (SAD), delta-12-fatty acid desaturase (FAD2) and omega-3-fatty acid desaturase (FAD3), respectively. Increased degree of unsaturation affects oxidative stability and ignition quality of the biodiesel [32]. Therefore, the high content of unsaturated fatty acids (78.4%) in jatropha oil is not desirable for biodiesel production. Knutzon *et al.*, 1992 [37] have significantly reduced the content of unsaturated fatty acids in transgenic *Brassica napus *by antisense expression of SAD gene. Complete removal of unsaturated fatty acid was not observed probably due to the presence of more than one SAD gene in *B. napus*. In our study, we found four different transcripts for SAD which could be used to design RNAi construct for efficient silencing of the expression of SAD enzyme in jatropha.

### Activation and transport of fatty acids

Free fatty acids that are released from acyl-ACPs are converted to respective acyl-CoAs (activation) by long chain acyl CoA synthetase (LCACS). The activated fatty acids are bound by acyl CoA binding proteins (ACBPs) to protect them from acyl-CoA hydrolases, and to transport them to endoplasmic reticulum. Arabidopsis contains nine LCACS genes [38] and six ACBP genes [39]. From the current study, we have identified four contigs for LCACS, and three of them were full-length. We also found five contigs for ACBPs, and two of them were full- length.

### Synthesis of triacylglycerol or oil

Triacyl glycerol or oil is synthesised in ER by serial incorporation of three acyl groups to the glycerol backbone. The first acyl group is added to the *sn-1 *position by glycerol-3-phosphate acyl transferase (GPAT). The resulting lysophosphatidic acid is further acylated at *sn-2 *position by lysophosphatidic acid acyl transferase (LPAT) to form phosphatidic acid. Phosphatidic acid is dephosphorylated by phosphatidic acid phosphatase (PAP) to form diacyl glycerol. Final acylation of diacyl glycerol in *sn-3 *position to complete the synthesis of triacylglycerol is carried out by diacyl glycerol acyl transferase (DGAT). These steps are highly amenable for genetic manipulations to increase the oil content. For example, seed oil content was increased by 21% in arabidopsis and 3-7% in canola by overexpressing GPAT [40] and DGAT [41], respectively. We have identified 12 contigs for the four enzymes, GPAT (4 contigs). LPAT (5 contigs), PAP (1 contig) and DGAT (2 contigs) that are required for triacyl glycerol synthesis. These sequences will be useful for increasing the oil content in jatropha by genetic engineering.

### Functional classification

**Figure 4:**
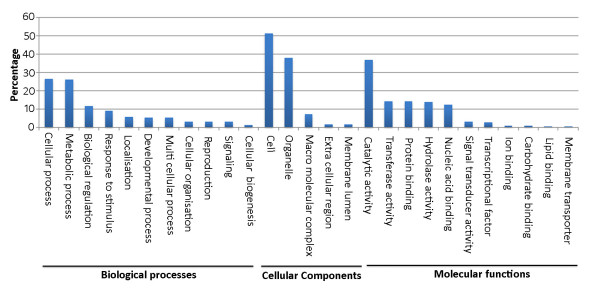
**Distribution of the 17,457 contigs under gene ontology terms: biological processes, cellular components, molecular functions**.

Gene ontology (GO) annotation and classification of the sequences was done based on three gene ontology terms such as biological processes, cellular components and molecular functions. Distribution of the contigs in different GO categories is given in figure 4. While the contigs covered all the major biological processes, the cellular and metabolic processes alone accounted for 52.7% of the contigs. Regulation of biological processes and response to stimulus accounted for 11.6% and 8.9% of the contigs, respectively. In cellular components, cell and organelles accounted for the maximum number of contigs (60%). In molecular functions, highest numbers of contigs were classified under catalytic activity (36.77%) which was followed by transferase activity, protein binding, hydrolase activity and nucleic acid binding activity (12.33 to 14.27%).

### Validation of assembled transcripts

**Figure 5:**
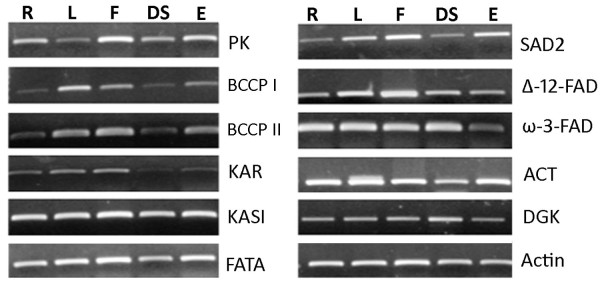
**Semi-quantitative RT-PCR analysis of the expression of eleven oil biosynthesis genes in Roots (R), mature leaves (L), flowers (F), developing seeds (DS), and embryos (E) of *J. curcas***. Name of the genes, primer sequences and PCR product size are given table 3.

**Figure 6:**
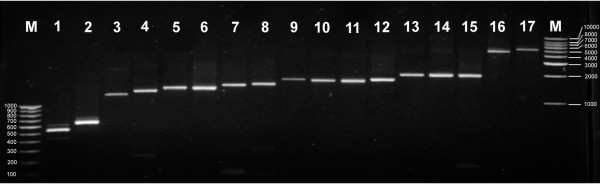
**RT-PCR amplification and agarose gel (1%) electrophoresis of seventeen full-length transcripts 1**. **ACBP, 2. ACP, 3. BCCPI, 4. BCCPII, 5. KAR, 6. FATA, 7. Δ-12 FAD, 8. ACT, 9. DGK, 10. KCS, 11. ω-3-FAD, 12. SAD, 13. KASI, 14. BC, 15. PK,16. PHD finger family protein, and 17. Kinase interacting family protein along with 1.0 kb DNA size markers (M1) and 100bp DNA size markers (M2)**. Name of the genes, primer sequences used, and the expected size of RT-PCR products are given in table 4.

The 3' untranslated region alone was RT-PCR amplified for 11 transcripts to validate the expression of assembled transcripts. All the transcripts were found to be expressed in all the five tissues studied (Figure 5). Whole transcripts were RT-PCR amplified for 17 transcripts to validate the expression and contig assembly. As shown in figure 6, all the transcripts were amplified successfully and were of expected size (500 to 5,000 bp approximately). In addition, *in silico *validation was done by comparing the assembled contig sequences with 7,009 high quality and manually edited ESTs and full-length cDNA sequences (100 and 2,372 bases) obtained from Sanger sequencing. We found 550 contigs to be fully mapping with the nucleotide sequence from Sanger sequencing, and the identity was 99.71% over a length of 100 to 1,932 bases. When 2,581 partially mapped contigs were analysed, the identity was 99.90% over a length of 37 to 1,900 bases. Overall, 1,253,655 bases were aligned with 99.87% identity. These results not only validate the accuracy of 454 pyrosequencing vis-à-vis Sanger sequencing but also the accuracy of contig assembly.

## Conclusions

We have analyzed the transcriptome from five major tissues of *J. curcas *using GS FLX titanium platform of 454 pyrosequencing for the purpose of large scale gene discovery. We assembled 17,457 contigs and identified 14,327 new transcripts to add to the existing jatropha genetic resource. Currently, 28,794 non-redundant transcripts sequences are available from jatropha. This includes 56 transcripts that are directly involved in oil biosynthesis. These sequences will be useful in genetic engineering of jatropha for increased oil content, modified oil composition, toxin-free oil cake, resistance to insect pests and diseases, tolerance to drought stress etc.

## Methods

### Collection of tissues for RNA extraction

We have used *Jatropha curcas *L. for our study. Tissue from roots was collected from two months old plants grown in a green house at 27 ± 2°C and 90% RH. Tissues from mature leaves, flowers, developing seeds, and embryo from mature dry seeds were collected from a jatropha plantation in Tamil Nadu, India. The tissues were immediately frozen in liquid nitrogen and stored at -86°C.

### RNA extraction

Total RNA from developing seeds was prepared using Trizol reagent as described before [10]. Total RNA from other tissues was isolated by following a protocol described by Singh *et al*., 2009 [42] except that the pellet obtained after lithium chloride precipitation was washed with 75% ethanol twice, and purified using RNeasy Mini Kit following the manufacturer's protocol (Qiagen, Hilden, Germany). Agarose gel electrophoresis and D_260_/OD_280 _ratio were used for assessing quality of total RNA.

### Synthesis of normalized cDNA

Pooled total RNA of 100 μg was prepared by mixing 5 μg, 25 μg, 15 μg, 30 μg, and 25 μg of total RNA from roots, mature leaves, flowers, developing seeds, and embryo, respectively. About 30 μg of the pooled total RNA was used for cDNAs synthesis and normalization was carried out as described by Patanjali *et al*., 1991 [43] and Soares *et al.*, 1994 [44] with slight modifications. Tester3 primer (CAGTGGTATCAACGCAGAGTGGCCGAGGCGGCCT_15_) and driver3 primer (GGGATAACAGGGTAATGGCCGAGGCGGCCGACATGT_15_) were used to prime first strand tester cDNA and driver cDNA synthesis, respectively. Tester adaptor (GTAACTAGGCCGTAATGGCCACTCTGCGTTGATACCACTG) and driver adaptor (GGCCGTAATGGCCTCGCTACCTTAGGA) were ligated to the 3' end of the first strand tester cDNA and driver cDNA, respectively. These adaptors were 5' phosphorylated and 3' blocked to prevent their ligation to the 5'end. Double strand tester cDNA was synthesised using tester3 primer and tester5 primer (CAGTGGTATCAACGCAGAGTGGCCATTACGGCCTAGTTACGGG). The tester5 primer is complementary to the 5' tester adaptor and it is phosphorylated. The sense strand of the double strand tester cDNA which is phosphorylated at the 5' end was destroyed by treating it with lambda exonuclease. As a result, only the antisense strands are retained. Double strand driver cDNA was synthesised using driver3 primer which is phosphorylated and driver5 primer (TCCTAAGGTAGCGAGGCCATTACGGCCGGG) which is complementary to the 5' driver adaptor. The anti-sense strand of the double strand driver cDNA which is phosphorylated at the 5' end was destroyed by treating it with lambda exonuclease. As a result, only the sense strands are retained. Hybridization of the anti-sense strands from tester cDNA and sense strands from driver cDNA was carried out in 1× hybridization buffer (50 mM Tris-HCl pH 8.0, 0.5 M NaCl, 0.2 mM EDTA) at 68°C for 6 h. Formation of double strand hybrids depends on the reassociation kinetics (second order kinetics), in which more abundant sequences anneal faster than rare sequences. Therefore, the double strand hybrids were removed by hydroxyapatite chromatography to achieve normalization [44]. The normalized single strand cDNAs were converted to double strand cDNAs and were amplified by Failsafe™ PCR system (Epicentre Biotechnologies, USA) using single amplification primer (CAGTGGTATCAACGCAGAGT) for which the binding sites are present only in the tester cDNA (underlined in tester3 and tester5 primers used for cDNA synthesis). Normalization efficiency was monitored by performing a parallel normalization in which chloramphenicol resistance gene was added to the cDNA at 1.0% redundant rate as internal control. Normalized cDNAs were cloned in modified pBluescript II SK^- ^and normalization efficiency was determined by plating the cDNA library on LB plates containing chloramphenicol.

### Quality Control of cDNA

An aliquot of normalized cDNA was used to construct a cDNA library in modified pBluescript II SK^- ^for quality control purpose. Ten colonies from the cDNA library were randomly selected and insert size was determined by colony PCR using M13 forward (GTAAAACGACGGCCAGT) and M13 reverse primer (CAGGAAACAGCTATGAC). Another 20 clones were randomly selected and sequenced from the 5' end of the cDNA using M13 reverse primer and BigDye™ Terminator v3.1 Cycle Sequencing Kit in 3130xl Genetic Analyzer (Applied Biosystems, CA, USA). These sequences were annotated by using BLASTX algorithm and non-redundant database at NCBI [45]. Full-length nature of the clones was determined from the BLASTX result as described before [10].

### 454 pyrosequencing

The normalized cDNA was quantified by PicoGreen assay and 3.8 μg of cDNA was used for making fragmentation library using GS FLX Titanium General Library Preparation Kit (Roche 454 Company, CT, USA) as described in the manufacturer's manual. Half-plate reaction of 454 pyrosequencing was done using 454 Genome Sequencer FLX System (Roche, CT, USA).

### *De novo *assembly

The 454 Genome Sequencer FLX System collects the data and generates standard flow gram file (.sff) which contains raw data for all the reads. The raw data was quality-filtered using a quality cut-off value of 40. The primer and adapter sequences that were incorporated during cDNA synthesis and normalization were removed. Sequences with less than 50bp were removed before contig assembly. *De Novo *contig assembly of the reads was performed using GS *De Novo *Assembler software versions, v2.3, v2.5p1 and v2.5.2 which were provided by 454 Life Sciences Corp, CT, USA. The assembly parameters used were minimum overlap length of 40 bp and minimum overlap identity of 95%.

### Pathway mapping using KEGG

Gene ortholog assignment and pathway mapping of the contigs was done using KEGG (Kyoto Encyclopaedia of Genes and Genomes) automatic annotation server [46]. The contigs were assigned with the unique enzyme commission (EC) numbers based on the similarity hit against KEGG database using BLASTX (default threshold bit-score value of 60)^. ^Distribution of contigs under the respective EC numbers was used to map them to the KEGG biochemical pathways.

### Annotation and functional classification

Annotation of the assembled transcript sequences was performed using BLASTX algorithm and non-redundant protein database at NCBI [45]. The BLASTX results were also used to assess the full-length nature of the contigs. The automated BLASTX analysis was done using BLAST2GO to assign GO terms for the contigs [47]. The transcripts were classified under three GO terms such as molecular function, cellular process and biological process.

### Semi-quantitative RT-PCR

For RT-PCR, total RNA from roots, mature leaves, flowers, developing seeds, and embryo were treated with DNase I and purified using RNeasy Mini Kit following the manufacturer's protocol (Qiagen, Hilden, Germany). About 3.0 μg of purified total RNA from each sample was used for first strand cDNA synthesis using oligo-dT_(18) _primer and PrimeScript™ reverse transcriptase (Takara Bio Inc, Shiga, Japan). Equal quantity of first strand cDNA (from 25 ng total RNA) was used for PCR. Primers were designed to amplify the 3' UTR of the transcripts. Actin gene was used as an internal control. Primer sequences and the expected size of the amplified fragments are given in table 3. Semi-quantitative analysis of the RT-PCR amplified fragments was done by agarose gel electrophoresis.

**Table 3:** Name of the genes, primers used and expected size of the RT-PCR products for semi-quantitative RT-PCR

S.No	Gene Name	Forward primer	Reverse primer	Product size (bp)
1	Biotin carboxyl carrier protein I of ACCase (BCCP I)	GTCGGCTAATCTTAAAGCTATTC	TGTTTATAGCTTCACTAGTGTAC	258
2	Biotin carboxyl carrier protein II of ACCase (BCCP II)	GATGCTCTCATTGCAATTCTC	TAATGATAAATAACAATGAAGAAGG	278
3	3-keto acyl ACP Reductase (KAR)	GTTATCTCTCCCGAAAGTGTA	CACAGTATCTGTCACCTTTTC	306
4	Beta-keto acyl ACP synthase I (KASI)	GCATCTGGCTTGTCTCCAT	GCAGAAGAACACAATTTTGATAC	402
5	Acyl ACP thioesterase (FATA)	AATAATGTAGATTTCTTTATTTGTGT	AGTAAACGTAAAACAATACAGTTGAT	285
6	ω-3-fatty acid Desaturase (ω-3-FAD)	AAAGCTGCAAAATTTTTTATCTGCA	CCCTCTCAAATCCAATCCAA	215
7	Diacyl glycerol kinase (DGK)	CGGCTATTCGGTTGGAAATAA	GATTTTTGATACAACAAATTACCAGT	296
8	Delta12-fatty acid desaturase(Δ-12 FAD)	CTGATCAAAGCAGAGGTGTGT	CTCTGAATTTGAATTTTTATAAACC	301
9	Acyl-CoA thioesterase (ACT2)	CACGTGGTTTTGTATCTGGC	ACCCCGGCTGCTAGAATCAA	277
10	Stearoyl-ACP desaturase (SAD)	ATCCAATGTGCCATTCAGCTG	ACCTCCTGCTCGAGAATAGA	419
11	Pyruvate kinase(PK)	GTCTCATGACAGGGTAGTTGT	ACCTGCCTGCCATGGCATTC	264
12	Actin	CAAGTCATCACCATTGGAGCA	CCTTGGAAATCCACATCTGTT	342

### RT-PCR amplification of full-length transcripts

First strand cDNA from 3.0 μg of pooled total RNA from the five tissues was prepared as described above, and entire length of 17 transcripts was amplified by PCR. Contig number, transcript (gene) name and primer sequences for RT-PCR are given in table 4. Size of the amplified fragments was determined by agarose gel electrophoresis using DNA markers.

**Table 4:** Name of the genes, primers used and expected size of the RT-PCR products for amplification of full-length transcripts

S.No	Gene Name	Forward primer	Reverse primer	Product size (bp)
1	Acyl-CoA binding protein (ACBP)	CCCGGATACGTTCTATAAATC	CCCACACGCAAGAGAAGACACTA	524
2	Acyl carrier protein (ACP)	GAACTCTCGCTACTCTCTTTC	GCATAAATTAGGAAATTTTAGAGTGT	641
3	Biotin carboxyl carrier protein I of ACCase (BCCP I)	CGGAAATTACAAAGACAGACAC	TGTTTATAGCTTCACTAGTGTAC	1220
4	Biotin carboxyl carrier protein II of ACCase (BCCP II)	GAGGTTTATGGAGTTGTAACTG	TAATGATAAATAACAATGAAGAAGGC	1316
5	3-keto acyl ACP Reductase (KAR)	ACAGACACATTCCTTTCTTCG	CACAGTATCTGTCACCTTTTC	1417
6	Acyl ACP thioesterase (FATA)	CTGACATTGGTCTTGAGCTCC	AGTAAACGTAAAACAATACAGTTGAT	1448
7	Delta12-fatty acid desaturase (Δ-12 FAD)	ACCGAGCAGAGGCCGGTATC	CTCTGAATTTGAATTTTTATAAACC	1501
8	Acyl-CoA thioesterase (ACT)	CCGGAGCCTGGACTACTGTT	ACCCCGGCTGCTAGAATCAA	1535
10	Diacyl Glycerol kinase (DGK)	CTATCTTCTTCAATACAAGTACG	GATTTTTGATACAACAAATTACCAGT	1709
9	3-keto acyl CoA synthase (KCS)	CCGATCAATATTGAAGCAAAT	ACCAGCACAAACAATCCCAG	1715
12	ω-3-fatty acid Desaturase (ω-3-FAD)	GAAGACAATAAGCACAAATTG	CCCTCTCAAATCCAATCCAA	1741
11	Stearoyl-ACP desaturase (SAD)	CCCACTTTGATGCGTCTGCT	GACCTCCTGCTCGAGAATAGA	1769
13	Beta-keto acyl ACP synthase I (KASI)	GTAGCTCAATTTGCTGCGCCT	GCAGAAGAACACAATTTTGATAC	1908
14	Biotin carboxylase subunit of heteromeric acetyl-CoA carboxylase (BC)	ACCGCATTCAATCTTTCTCAC	GACTTCATCAACTCACTTCTA	1944
15	Pyruvate Kinase (PK)	ACCGAGGTTTCGGTCTTCACC	GACCTGCCTGCCATGGCATTC	1974
16	PHD finger family protein	ATCGTGTTCAATTTAGGATTGAG	TCTGGAGTCTTGTTCTGGGTA	4512
17	Kinase interacting family protein	GTACCTTATGTTCTGAATGATGA	TGAAGTCCTTCAAGATGACAC	5030

### Accession number

All the reads generated and used for this study were submitted to the sequence read archive (SRA) at NCBI with the accession number SRP004898.

## Competing interests

The authors declare that they have no competing interests.

## Authors' contributions

The study was conceived and directed by MP. All the experiments and analysis were directed by MP and carried out by PN. MP and PN wrote the paper. Both authors read and approved the final manuscript.

## Supplementary Material

Additional file 1 **Total RNA isolation and normalized cDNA library construction**. Total RNA was isolated from roots (R), mature leaves (L), flowers (F), developing seeds (DS), and embryos (E) of *Jatropha curcas *(Figure A). Normalized cDNA library was constructed from pooled total RNA and the cDNA inserts were PCR amplified from 10 randomly selected clones and resolved in 1.0% agarose gel electrophoresis with 1.0 kb DNA size markers (Figure B). Click here for file

Additional file 2 **Reference mapping statistics**. Reference assembly with the partial genomic sequence of jatropha showed mapping of 95.87% of the reads and consensus accuracy was 99.15%. Click here for file

Additional file 3 **Contigs from *de novo *assembly of 383,918 reads from the current study**. The contig sequences obtained from the *de novo *assembly of 3, 83,918 reads from *Jatropha curcas *were given as FASTA format in TXT file. Click here for file

Additional file 4 **Contigs from *de novo *assembly of 991,050 reads from *Sato et al.*, 2011 **[9]. The contig sequences obtained from the *de novo *assembly of 991,050 reads from Sato *et al.*, 2011 [9] were given as FASTA format in TXT file. Click here for file

## References

[B1] Mabberley DJ The Plant Book, A portable dictionary of vascular plants Cambridge University Press 2005 Cambridge

[B2] Openshaw K A review of *Jatropha curcas*: an oil plant of unfulfilled promise Biomass Bioenerg 2000 19 1 15 10.1016/S0961-9534(00)00019-2

[B3] Lin J Yan F Tang L Chen F Antitumor effects of curcin from seeds of *Jatropha curcs* Acta Pharmacol Sin 2003 24 241 6 12617773

[B4] Igbinosa OO Igbinosa EO Aiyegoro OA Antimicrobial activity and phytochemical screening of stem bark extracts from *Jatropha curcas *(Linn) Afr J Pharm Pharacol 2009 3 058 062

[B5] Fagbenro-Beyioku AF Oyibo WA Anuforom BC Disinfectant/antiparasitic activities of *Jatropha curcas* East Africa Med. J 1998 75 508 511 10493051

[B6] Mujumdar AM Misar AV Salaskar MV Upadhye AS Antidiarrhoeal effect of an isolated fraction (JC) of *Jatropha curcas *roots in mice J. Nat. Remedies 2001 1 89 93

[B7] Carvalhoa CR Clarindoa WR Praca MM Araujo FS Carels N Genome size, base composition and karyotype of Jatropha curcas L., an important biofuel plant Plant Sci 2008 174 613 617 10.1016/j.plantsci.2008.03.010

[B8] Synthetic genomics http://www.syntheticgenomics.com/media/press/52009.html

[B9] Sato S Hirakawa H Isobe S Fukai E Watanabe A Kato M Kawashima K Minami C Muraki A Nakazaki N Takahashi C Nakayama S Kishida Y Kohara M Yamada M Tsuruoka H Sasamoto S Tabata S Aizu A Toyoda A Shin-i T Minakuchi Y Kohara Y Fujiyama A Tsuchimoto S Kajiyama S Makigano E Ohmido N Shibagaki N Cartagena JA Wada N Kohinata T Atefeh A Yuasa S Matsunaga S Fukui K Sequence Analysis of the Genome of an Oil-Bearing Tree, *Jatropha curcas *L DNA Res 2011 18 65 76 10.1093/dnares/dsq030 21149391 PMC3041505

[B10] Natarajan P Kanagasabapathy D Gunadayalan G Panchalingam J Shree N Sugantham PA Singh KK Madasamy P Gene discovery from *Jatropha curcas *by sequencing of ESTs from normalized and full-length enriched cDNA library from developing seeds BMC Genomics 2010 11 606 10.1186/1471-2164-11-606 20979643 PMC3091748

[B11] Costa GGL Cardoso KC Del Bem LEV Lima AC Cunha MAS Campos-Leite LD Vicentini R Papes F Moreira RC Yunes JA Campos FAP Silva MJD Transcriptome analysis of the oil-rich seed of the bioenergy crop Jatropha curcas L BMC Genomics 2010 11 462 10.1186/1471-2164-11-462 20691070 PMC3091658

[B12] Weber APM Weber KL Carr K Wilkerson C Ohlrogge JB Sampling the arabidopsis transcriptome with massively parallel pyrosequencing Plant Physiol 2007 144 32 42 10.1104/pp.107.096677 17351049 PMC1913805

[B13] Wang W Wang Y Zhang Q Qi Y Guo D Global characterization of *Artemisia annua *glandular trichome transcriptome using 454 pyrosequencing BMC Genomics 2009 10 465 10.1186/1471-2164-10-465 19818120 PMC2763888

[B14] Guo S Zheng Y Joung JG Liu S Zhang Z Crasta OR Sobral BW Xu Y Huang S Fei Z Transcriptome sequencing and comparative analysis of cucumber flowers with different sex types BMC Genomics 2010 11 384 10.1186/1471-2164-11-384 20565788 PMC2897810

[B15] Cheung F Haas BJ Goldberg SMD May GD Xiao Y Town CD Sequencing *Medicago truncatula *expressed sequenced tags using 454 Life Sciences technology BMC Genomics 2006 7 272 10.1186/1471-2164-7-272 17062153 PMC1635983

[B16] Arreguín JCV Laclette EI Moraila BJ Martínez O Calzada JPV Estrella LH Estrella AH Deep sampling of the *Palomero *maize transcriptome by a high throughput strategy of pyrosequencing BMC Genomics 2009 10 299 19580677 10.1186/1471-2164-10-299 PMC2714558

[B17] Wicker T Schlagenhauf E Graner A Close TJ Keller B Stein N 454 sequencing put to the test using the complex genome of barley BMC Genomics 2006 7 275 10.1186/1471-2164-7-275 17067373 PMC1633745

[B18] Vera JC Wheat CW Fescemyer HW Frilander MJ Crawford DL Hanski I Marden JH Rapid transcriptome characterization for a non model organism using 454 pyrosequencing Mol Ecol 2008 17 1636 1647 10.1111/j.1365-294X.2008.03666.x 18266620

[B19] Cheung F Win J Lang JM Hamilton J Vuong H Leach JE Kamoun S Levesque CA Tisserat N Buell CR Analysis of the *Pythium ultimum *transcriptome using Sanger and Pyrosequencing approaches BMC Genomics 2008 9 542 10.1186/1471-2164-9-542 19014603 PMC2612028

[B20] Sun C Li Y Wu Q Luo H Sun Y Song J Lui EMK Chen S *De novo*le sequencing and analysis of the American ginseng root transcriptome using a GS FLX Titanium platform to discover putative genes involved in ginsenoside biosynthesis BMC Genomics 2010 11 262 10.1186/1471-2164-11-262 20416102 PMC2873478

[B21] Kumar S Blaxter ML Comparing de novo assemblers for 454 transcriptome data BMC Genomics 2010 11 571 10.1186/1471-2164-11-571 20950480 PMC3091720

[B22] The Arabidopsis Lipid Gene Database http://www.lipids.plantbiology.msu.edu/

[B23] Lin M Behal R Oliver DJ Disruption of *plE2*, the gene for the E2 subunit of the plastid pyruvate dehydrogenase complex, in *Arabidopsis *causes an early embryo lethal phenotype Plant Mol Biol 2003 52 865 872 10.1023/A:1025076805902 13677473

[B24] Konishi T Shinohara K Yamada K Sasaki Y Acetyl-CoA carboxylase in higher plants; most plants other than Gramineae have both the prokaryotic and the eukaryotic forms of this enzyme Plant Cell Physiol 1996 37 117 122 8665091 10.1093/oxfordjournals.pcp.a028920

[B25] Ke J Wen TN Nikolau BJ Wurtele ES Coordinate Regulation of the Nuclear and Plastidic Genes Coding for the Subunits of the Heteromeric Acetyl-Coenzyme A Carboxylase Plant Physiol 2000 122 1057 1072 10.1104/pp.122.4.1057 10759501 PMC58940

[B26] Madoka Y Tomizawa KI Mizoi J Nishida I Nagano Y Sasaki Y Chloroplast Transformation with Modified *accD *Operon Increases Acetyl-CoA Carboxylase and Causes Extension of Leaf Longevity and Increase in Seed Yield in Tobacco Plant Cell Physiol 2002 43 1518 1525 10.1093/pcp/pcf172 12514249

[B27] Nakkaew A Chotigeat W Eksomtramage T Phongdara A Cloning and expression of a plastid-encoded subunit, beta-carboxyltransferase gene (*accD*) and a nuclear-encoded subunit, biotin carboxylase of acetyl-CoA carboxylase from oil palm (*Elaeis guineensis *Jacq.) Plant Sci 2008 175 497 504 10.1016/j.plantsci.2008.05.023

[B28] Klaus D Ohlrogge JB Neuhaus HE Dormann P Increased fatty acid production in potato by engineering of acetyl-CoA carboxylase Planta 2004 219 389 396 10.1007/s00425-004-1236-3 15014998

[B29] Roesler K Shintani D Savage L Boddupalli S Ohlrogge J Targetting of the Arabidopsis homomeric acetyl-Coenzyme A carboxylase to plastids of rapeseeds Plant Physiol 1997 113 75 81 10.1104/pp.113.1.75 9008389 PMC158117

[B30] Wu GZ Xue HW *Arabidopsis *β-Ketoacyl-[Acyl Carrier Protein] Synthase I Is Crucial for Fatty Acid Synthesis and Plays a Role in Chloroplast Division and Embryo Development Plant Cell 2010 22 3726 3744 10.1105/tpc.110.075564 21081696 PMC3015132

[B31] Allen CAW Watts KC Ackman RG Pegg MJ Predicting the viscosity of biodiesel fuels from their fatty acid ester composition Fuel 1999 78 1319 1326 10.1016/S0016-2361(99)00059-9

[B32] Knothe G Dependence of biodiesel fuel properties on the structure of fatty acid alkyl esters Fuel Processing Technology 2005 86 1059 1070 10.1016/j.fuproc.2004.11.002

[B33] Akbar E Yaakob Z Kamarudin SK Ismail M Salimon J Characteristic and Composition of *Jatropha Curcas *Oil Seedfrom Malaysia and its Potential as Biodiesel Feedstock European Journal of Scientific Research 2009 29 396 403

[B34] Aghoram K Wilson RF Burton JW Dewey RE A Mutation in a 3-Keto-Acyl-ACP synthase II gene is associated with elevated palmitic acid Levels in soybean seeds Crop Sci 2006 46 2453 2459 10.2135/cropsci2006.04.0218

[B35] Nguyen T Shanklin J Altering arabidopsis oilseed composition by a combined antisense-hairpin RNAi gene suppression Approach J Am Oil Chem Soc 2009 86 41 49 10.1007/s11746-008-1322-y

[B36] Dormann P Voelker TA Ohlrogge JB Accumulation of palmitate in arabidopsis mediated by the acyl-Acyl carrier protein thioesterase FATB1 Plant Physiol 2000 123 637 643 10.1104/pp.123.2.637 10859193 PMC59031

[B37] Knutzon DS Thompson GA Radke SE Johnson WB Knauf VC Kridl JC Modification of Brassica seed oil by antisense expression of a stearoyl-acyl carrier protein desaturase gene Proc Natl Acad Sci 1992 89 2624 2628 10.1073/pnas.89.7.2624 1557366 PMC48714

[B38] Shockey JM Fulda MS Browse JA *Arabidopsis *Contains Nine Long- chain acyl-Coenzyme A synthetase genes that participate in fatty acid and glycerolipid metabolism Plant Physiol 2002 129 1710 1722 10.1104/pp.003269 12177484 PMC166759

[B39] Xiao S Chye ML An Arabidopsis family of six acyl-CoA-binding proteins has three cytosolic members Plant Physiol Biochem 2009 47 479 84 10.1016/j.plaphy.2008.12.002 19121948

[B40] Jain RK Coffey M Lai K Kumar A MacKenzie SL Enhancement of seed oil content by expression of glycerol-3-phosphate acyltransferase genes Biochem Soc Trans 2000 28 958 61 10.1042/BST0280958 11171271

[B41] Taylor DC Zhang Y Kumar A Francis T Giblin EM Barton DL Ferrie JR Laroche A Shah S Zhu W Snyder CL Hall L Rakow G Harwood JL Weselake RJ Molecular modification of triacylglycerol accumulation by over-expression of *DGAT1 *to produce canola with increased seed oil content under field conditions Botany 2009 87 533 543 10.1139/B08-101

[B42] Singh RK Misra A Sane VA Nath P Isolation of high Quality RNA from oilseeds of *Jatropha curcas* J. Plant Biochem. Botechnol 2009 18 77 81

[B43] Patanjali SR Parimoo S Weissman SM Construction of a uniform-abundance (normalized) cDNA library Proc Natl Acad Sci 1991 88 1943 1947 10.1073/pnas.88.5.1943 1705712 PMC51142

[B44] Soares MB Bonaldo MF Jelene P Su L Lawton L Efstratiadis A Construction and characterization of a normalized cDNA library Proc Natl Acad Sci 1994 91 9228 9232 10.1073/pnas.91.20.9228 7937745 PMC44785

[B45] National Centre for Biotechnology Information BLAST http://blast.ncbi.nlm.nih.gov/Blast.cgi

[B46] Moriya Y Itoh M Okuda S Yoshizawa A Kanehisa M KAAS: an automatic genome annotation and pathway reconstruction server Nucleic Acids Res 2007 35 W182 W185 10.1093/nar/gkm321 17526522 PMC1933193

[B47] A universal gene ontology annotation, visualization and analysis tool for functional genomics research version 2.4.5 http://www.blast2go.org/

