# Supplementary figures and images for: De novo assembly and transcriptome analysis of five major tissues of Jatropha curcas L. using GS FLX titanium platform of 454 pyrosequencing
Source: BMC Genomics. 2011 Apr 15;12:191. doi: 10.1186/1471-2164-12-191 (PMC3087711; doi:10.1186/1471-2164-12-191)

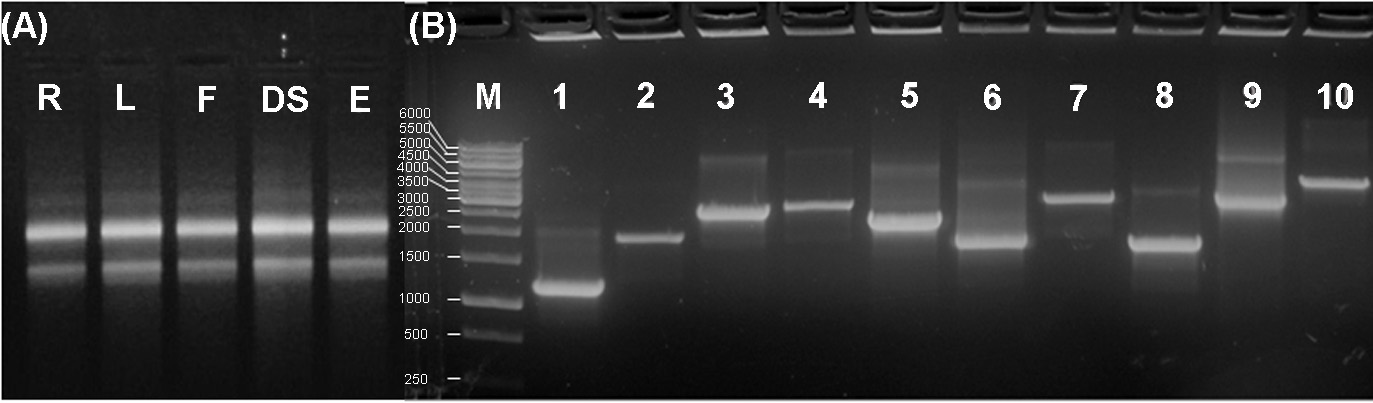

Supplement: Additional file 1 — Total RNA isolation and normalized cDNA library construction. Total RNA was isolated from roots (R), mature leaves (L), flowers (F), developing seeds (DS), and embryos (E) of Jatropha curcas (Figure A). Normalized cDNA library was constructed from pooled total RNA and the cDNA inserts were PCR amplified from 10 randomly selected clones and resolved in 1.0% agarose gel electrophoresis with 1.0 kb DNA size markers (Figure B). [file 1471-2164-12-191-S1.JPEG]
