# Supplementary material for: De novo assembly and transcriptome analysis of five major tissues of Jatropha curcas L. using GS FLX titanium platform of 454 pyrosequencing
Source: BMC Genomics. 2011 Apr 15;12:191. doi: 10.1186/1471-2164-12-191 (PMC3087711; doi:10.1186/1471-2164-12-191)
Supplement: Additional file 2 — Reference mapping statistics. Reference assembly with the partial genomic sequence of jatropha showed mapping of 95.87% of the reads and consensus accuracy was 99.15%. [file 1471-2164-12-191-S2.DOC]

**Reference mapping statistics**

| Number of reference sequences | 150,417 |
| --- | --- |
| Number of bases in the reference sequence | 285,858,490 |
| Number of reads subjected for mapping | 383,918 |
| Number of reads used for mapping | 379,655 |
| Number of reads mapped | 363,987 |
| Number of reads fully mapped | 122,955 |
| Number of reads partially mapped | 68,319 |
| Number of reads mapped as chimeric | 172,713 |
| Number of reads unmapped | 15,668 |
| Percentage of the reads mapped | 95.87 |
| Number of bases mapped | 111,239,571 |
| Percentage of bases mapped | 88.62 |
| Percentage of consensus accuracy | 99.15 |
